# Caspase‐3‐mediated GSDME activation contributes to cisplatin‐ and doxorubicin‐induced secondary necrosis in mouse macrophages

**DOI:** 10.1111/cpr.12663

**Published:** 2019-07-26

**Authors:** Feng‐Yi Mai, Pengyan He, Jie‐Zhou Ye, Li‐Hui Xu, Dong‐Yun Ouyang, Chen‐Guang Li, Qiong‐Zhen Zeng, Chen‐Ying Zeng, Cheng‐Cheng Zhang, Xian‐Hui He, Bo Hu

**Affiliations:** ^1^ Department of Immunobiology, College of Life Science and Technology Jinan University Guangzhou China; ^2^ School of Medicine Sun Yat‐Sen University Shenzhen China; ^3^ Department of Cell Biology, College of Life Science and Technology Jinan University Guangzhou China; ^4^ Department of Nephrology the First Affiliated Hospital of Jinan University Guangzhou China

**Keywords:** caspase‐3, chemotherapeutic drugs, gasdermin E, macrophages, secondary necrosis

## Abstract

**Objective:**

Induction of secondary necrosis/pyroptosis contributes to the toxicity of chemotherapeutic drugs**,** in which gasdermin E (GSDME) plays critical roles. This study aimed to explore whether GSDME is involved in mediating the cytotoxic effects of cisplatin and doxorubicin on mouse macrophages.

**Methods:**

RAW 264.7 cells and bone marrow‐derived macrophages (BMDMs) were treated with cisplatin or doxorubicin. Propidium iodide staining was used to assay necrosis, and immunoblotting was performed to detect protein expression. GSDME was knocked down by using small interfering RNA. Mice were injected intraperitoneally to evaluate toxicity to macrophages in vivo. Flow cytometry and immunofluorescence microscopy were adopted to analyse phenotypes of peritoneal cells**.** Cytokine levels were assayed by cytometric bead array.

**Results:**

Both cisplatin and doxorubicin dose‐dependently induced necrosis in mouse RAW 264.7 macrophages and BMDMs. Accompanying this, multiple caspases were activated, concomitant with the cleavage of poly (ADP‐ribose) polymerase. Consistent with caspase‐3 activation, GSDME was cleaved to generate its N‐terminal fragment (GSDME‐NT), thus leading to secondary necrosis/pyroptosis. Inhibition of caspase‐3 significantly attenuated the generation of GSDME‐NT concurrently with decreased necrosis in macrophages. GSDME knockdown also evidently decreased the necrosis in RAW 264.7 and BMDMs. Besides, cisplatin administration depleted peritoneal macrophages in mice, which was associated with caspase‐3 activation and GSDME‐NT generation. Consistent with the macrophage depletion, cisplatin administration significantly decreased survival of mice with bacterial infection.

**Conclusion:**

Chemotherapeutic cisplatin and doxorubicin exerted their cytotoxicity on macrophages partly by inducing caspase‐3/GSDME‐mediated secondary necrosis.

## 1 INTRODUCTION

DNA‐damaging agents, including cisplatin and doxorubicin, have been used for cancer chemotherapy for decades and are currently still the mainstays of chemotherapeutic drugs. They are classified into two categories by modes of action: DNA cross‐linkers and topoisomerase II poisons.^1^ Among them, cisplatin (cis‐diamminedichloroplatinum II) is a representative DNA cross‐linking agent, while doxorubicin belongs to anthracyclines that can poison topoisomerase II.^2^, ^3^ All these chemotherapeutic drugs can damage DNA and thus cause cell‐cycle arrest in cancer cells. Compared with normal cells, cancer cells are more susceptible to DNA damage due to their relaxed DNA damage sensing/repair abilities and high proliferation rate. Therefore, DNA‐damaging agents are widely used in treating various solid cancers and leukaemia.^1^ Concurrent with their therapeutic effects on cancer cells, however, chemotherapeutic drugs have adverse toxicity to normal tissues. For example, cisplatin has the major side effects of nephrotoxicity, neurotoxicity and ototoxicity, while doxorubicin can cause cardiotoxicity, myelosuppression and neurotoxicity.^1^ Uncovering the mechanisms for the adverse toxicity may improve the application of DNA‐damaging agents in clinic.

Mechanistically, DNA‐damaging chemotherapeutic drugs, including cisplatin and doxorubicin, have been shown to suppress tumour growth by inducing cancer cell apoptosis.^2^, ^3^ Both extrinsic and intrinsic apoptosis pathways can be triggered to activate caspase‐8 and caspase‐9, respectively, which in turn activate their downstream caspase‐3/caspase‐7 leading to apoptotic cell death.^4^ Recent studies have revealed that activated caspase‐3 can also cleave gasdermin E (GSDME) to generate its N‐terminal fragment (GSDME‐NT), which executes secondary necrosis/pyroptosis by forming pores in the plasma membrane.^5^, ^6^ Upon apoptosis stimulation, cells lacking GSDME expression undergo apoptosis without progression into necrosis, whereas those with high or moderate GSDME levels directly undergo GSDME‐mediated pyroptosis.^6^ It is therefore believed that GSDME expression may govern the transition between apoptosis and necrosis/pyroptosis.^5^ However, the expression of GSDME in many cancer cells has been down‐regulated or even silenced due to epigenetic modification at its promoter region.^7^, ^8^ In contrast, GSDME is highly expressed in a variety of normal tissues, such as lungs, kidney, testis and placenta; it is also moderately expressed in the heart, pancreas, stomach, small intestines and brain.^5^, ^7^, ^9^ Importantly, cisplatin administration caused severe injury in the small intestines and lungs of wild‐type mice, whereas such tissue injury was partially prevented in *GSDME* ^−/−^ mice.^5^ This suggests that anti‐cancer chemotherapy can do harm to normal tissues of patients, leading to severe side effects.

Macrophages are important innate immune cells acting as sentinels in tissues against pathogenic infections.^10^ GSDME has been shown to be expressed in macrophages.^6^ It remains incompletely understood whether chemotherapeutic drugs do harm to macrophages by inducing GSDME‐mediated necrosis/pyroptosis. We found in this study that cisplatin and doxorubicin induced secondary necrosis in mouse macrophages in vitro and in vivo*,* in part through the caspase‐3/GSDME axis. Such cytotoxicity was involved in depleting peritoneal macrophages with recruitment of inflammatory monocytes. Mice administered with cisplatin had drastically increased susceptibility to bacterial infection. These results suggest that chemotherapeutic agents exert adverse toxicity on macrophages partly by inducing GSDME‐mediated secondary necrosis, thus compromising the innate immunity against infections.

## 2 MATERIALS AND METHODS

### 2.1 Reagents and antibodies

Cisplatin, doxorubicin and VX‐765 were purchased from Selleck. Ac‐DEVD‐CHO was obtained from MedChem Express. Propidium iodide (PI), Hoechst 33342, dimethyl sulfoxide (DMSO) and Tween‐20 were bought from Sigma‐Aldrich. Lipofectamine RNAiMAX, Dulbecco's Modified Eagle's Medium (DMEM) medium with high glucose, Opti‐MEM, foetal bovine serum (FBS), streptomycin and penicillin were the products of ThermoFisher. The antibody against actin was purchased from Santa Cruz. The antibodies against cleaved caspase‐3, caspase‐3, cleaved caspase‐7, cleaved caspase‐8, cleaved caspase‐9, PARP and horse‐radish peroxidase (HRP)‐linked IgG were purchased from Cell Signaling Technology. Antibodies against pro‐caspase1+p10+p12, GSDMD (clone: EPR19828) and DFNA5/GSDME were obtained from Abcam. F4/80‐PE, Ly‐6C‐APC and CD11b‐AlexaFluor488 were obtained from eBioscience. F4/80‐AlexaFluor647 was bought from BioLegend.

### 2.2 Animals

C57BL/6 mice (6‐8 weeks of age) were bought from the Experimental Animal Center of Southern Medical University (Guangzhou, China). All animals were acclimatized for 1 week before experiments and maintained under a 12‐hours/12‐hours dark/light cycle condition and had free access to water and food. All animal experiments were performed according to the guidelines for the care and use of animals approved by the Committee on the Ethics of Animal Experiments of Jinan University.

### 2.3 Cell culture

Mouse macrophage cell line RAW 264.7 cells were obtained from the Cell Bank of the Chinese Academy of Sciences (Shanghai, China) and maintained in complete DMEM medium (containing 10% FBS, 100 IU/mL penicillin, 100 µg/mL streptomycin and 2 mmol/L L‐glutamine) and cultured at 37°C in a humidified incubator with 5% CO_2_. Bone marrow‐derived macrophages (BMDMs) from mice were differentiated as previously described.^11^, ^12^

### 2.4 Cell death assay

Cell death was measured by PI incorporation as described previously.^12^, ^13^ Cells were treated with indicated concentrations of cisplatin in complete DMEM medium for 16 hours, stained with PI solution (2 µg/mL PI) and observed immediately by live imaging using Zeiss Axio Observer D1 microscope (Carl Zeiss).

### 2.5 Western blot analysis

Western blotting was performed essentially as previously described.^12^ Briefly, total proteins were separated by sodium dodecyl sulphate‐polyacrylamide gel electrophoresis (SDS‐PAGE) and electro‐transferred to PVDF membranes (Roche). After blocking, the membranes were incubated with indicated primary antibody and HRP‐linked IgG. Bands were revealed with enhanced chemiluminescence and recorded by X‐ray films. The blot images were captured by FluorChem 8000 imaging system (Alpha Innotech). The grey values were analysed by AlphaEaseFC 4.0 (Alpha Innotech).

### 2.6 Small interfering RNA (siRNA)

The siRNA (5′‐GCTGCAAACTCCATGTTAT‐3′) duplexes targeting mouse GSMDE/DFNA5 and negative control (NC) siRNA were designed and synthesized by RiboBio. Transfection was performed using Lipofectamine RNAiMAX according to the instructions provided by the manufacturer. In brief, RAW 264.7 cells and BMDMs were plated in 24‐well plates and transfected with GSDME siRNA (20 nmol/L) or NC siRNA. The cells were cultured in DMEM medium containing 10% FBS for 48 hours, followed by treatment with cisplatin.

### 2.7 Bacterial infection

The mouse model of bacterial infection was performed as previously described.^12^, ^14^ Briefly, after acclimated for 1 week, mice were intraperitoneally (ip) administered with cisplatin (20 and 50 mg/kg body weight) or vehicle (PBS) once. Three hours later, mice were injection (ip) with freshly prepared, viable *Escherichia coli* (DH5α) cells (1.5 × 10^9^ colony‐forming units (CFU)/mouse) in 0.5 mL PBS. Mouse survival was monitored every 6 hours for five consecutive days. In a separate experiment, mice were injected (ip) with cisplatin (20 mg/kg body weight) or vehicle. After 16 hours, mice were anesthetized and sacrificed. Their serum, peritoneal exudate cells and peritoneal lavage fluids were collected.

### 2.8 Immunofluorescence microscopy

Immunofluorescence analysis was performed as previously described.^12^ In brief, peritoneal exudate cells were seeded in glass‐bottomed dishes (1.5 × 10^5^ cells/dish). After fixation, permeabilization and blocking, cells were incubated with CD11b‐AlexaFluor488, F4/80‐AlexaFluor647 antibodies overnight. After staining with Hoechst 33342 solution (5 µg/mL in PBS) to reveal the nuclei, the cells were observed under a Zeiss Axio Observer D1 microscope (Carl Zeiss).

### 2.9 Flow cytometry

For phenotyping analysis, peritoneal exudate cells were collected and washed with PBS‐F (PBS containing 0.1% NaN_3_ and 3% FBS), followed by staining with CD11b‐FITC, F4/80‐PE and Ly‐6C‐APC at 4°C for 30 minutes. Red blood cells, if there were, were lysed. After washing with PBS‐F, cells were fixed with 4% paraformaldehyde in PBS and then analysed on a flow cytometer (Attune NxT; ThermoFisher). Cytokines in peritoneal lavage fluids were measured by flow cytometry together with cytometric bead array (CBA) mouse inflammation kit (BD Biosciences) according to the instructions of manufacturer. Data were acquired and analysed by using the Attune NxT software (ThermoFisher).

### 2.10 Statistical analysis

All experiments were performed three times independently, with one representative experiment shown. The data were expressed as mean ± standard deviation (SD) and analysed for statistical significance using GraphPad Prism 5.0 (GraphPad Software Inc). One‐way analysis of variance (ANOVA) followed by Dunnett post hoc test and unpaired Student's *t* test was used to analyse the statistical significance among multiple groups and between two groups, respectively. Kaplan‐Meier survival curves were adopted for analysis of mouse survival, and the statistical difference between two groups was determined using the log‐rank (Mantel‐Cox) test. *P*‐values <.05 were considered statistically significant.

## 3 RESULTS

### 3.1 Cisplatin and doxorubicin induced necrosis in mouse macrophages

**Figure 1:**
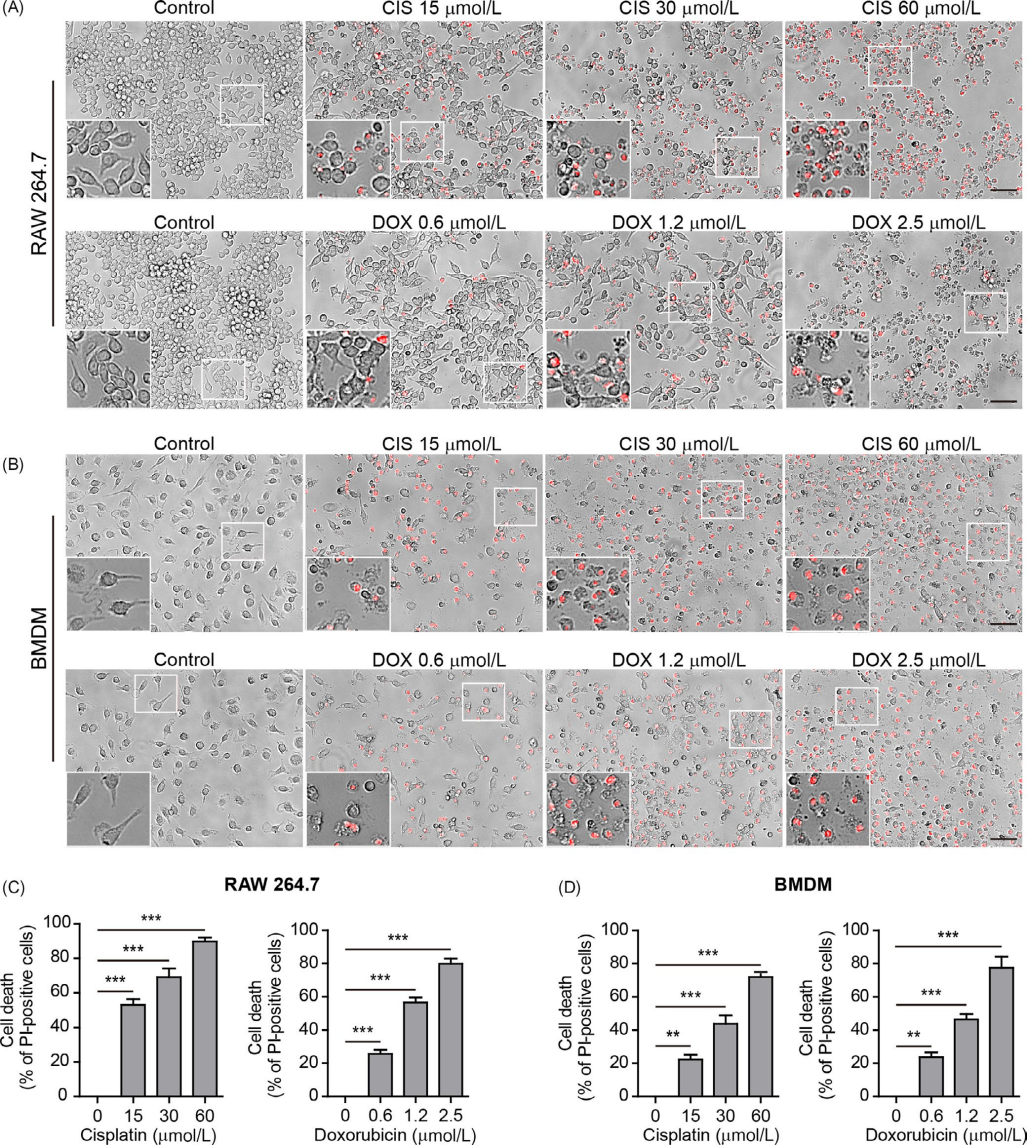
Cisplatin‐ and doxorubicin‐induced necrosis in mouse macrophages in culture. A, RAW 264.7 cells were treated with graded concentrations of cisplatin and doxorubicin for 16 h. Lytic cell death (necrosis) was assayed by propidium iodide (PI) staining (positive staining for dying cells). Images were captured by fluorescence microscopy, merged with bright‐field ones. One set of representative images of three independent experiments are shown. Scale bars, 50 μm. B, Representative images showing necrosis in bone marrow‐derived macrophages (BMDMs) treated as in (A). The inset represents a magnified area of each image. C,D, Quantification of PI‐positive cells in 5 randomly chosen fields each containing ~100 cells in (A) and (B), respectively. Data are shown as mean ± SD (n = 5). **P < .01; ***P < .001

To explore the cytotoxic effects of chemotherapeutic agents on macrophages, we used propidium iodide (PI) staining to evaluate the necrosis in RAW 264.7 cells and primary BMDMs. Incorporation of PI into cells is indicative of loss of membrane integrity and thus characterizing lytic cell death (necrosis). The results showed that both cisplatin and doxorubicin had induced cell rounding, shrinkage and fragmentation, concomitant with PI incorporation into those cells (Figure 1 A,B), suggesting apoptosis concurrent with necrosis. Quantitative analysis revealed that cisplatin and doxorubicin dose‐dependently induced necrosis in both RAW 264.7 cells and BMDMs (Figure 1 C,D). These results suggested cisplatin and doxorubicin were able to induce necrosis in mouse macrophages.

### 3.2 Multiple caspase pathways and GSDME were activated in macrophages by cisplatin or doxorubicin

**Figure 2:**
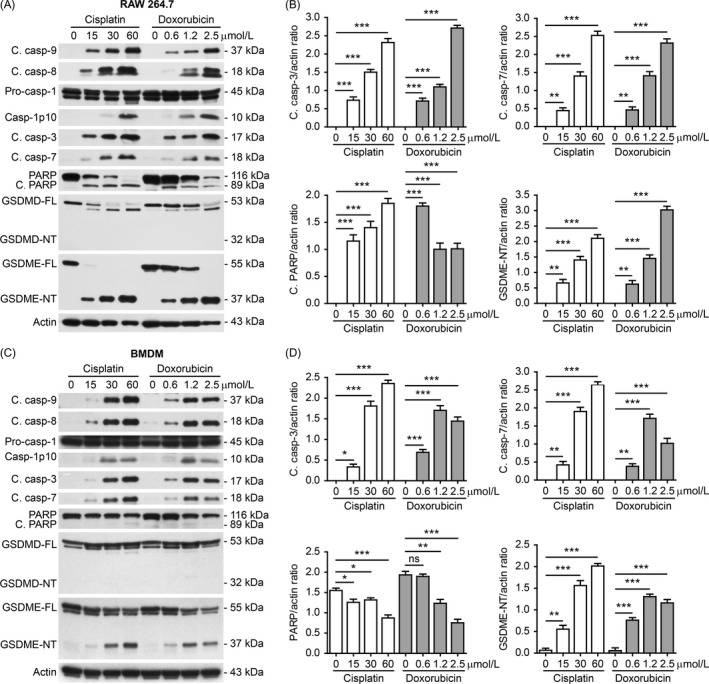
Multiple caspase pathways were activated in mouse macrophages in response to cisplatin and doxorubicin. A, RAW 264.7 cells were treated with graded concentrations of cisplatin and doxorubicin for 16 h. Western blot analysis of indicated proteins in the cell lysates was performed. Actin was recruited as a loading control. B, Western blotting of indicated proteins in bone marrow‐derived macrophages (BMDMs) treated as in (A). C,D, The ratios of indicated proteins in (A) and (B) were quantified relatively to their respective actin by densitometry. Data are shown as mean ± SD (n = 3). *P < .05; **P < .01; ***P < .001; ns, not significant; GSDME‐NT, GSDME N‐terminal fragment; GSDME‐FL, full‐length GSDME

We next assessed whether apoptotic caspases and inflammatory caspases were activated in the macrophages upon cisplatin or doxorubicin exposure. Western blot analysis revealed that both apoptosis initiator caspases (caspase‐8/caspase‐9) and executioner caspases (caspase‐3/caspase‐7) were activated in a dose‐dependent manner by cisplatin or doxorubicin in both RAW 264.7 cells and BMDMs (Figure 2 A‐D). Consistent with caspase‐3/caspase‐7 activation, their conventional substrate poly (ADP‐ribose) polymerase (PARP) was cleaved to generate an 89 kDa fragment in RAW 264.7. In BMDMs, the full‐length PARP was decreased by the drugs although the cleaved PARP bands were hardly detectable (Figure 2 A‐D). Unexpectedly, cleaved caspase‐1p10 (indicative of caspase‐1 activation) was synchronously detected in RAW 264.7 and BMDMs treated with cisplatin or doxorubicin (Figure 2 A,C). These results indicated that both apoptotic and inflammatory caspase pathways were activated in mouse macrophages upon cisplatin or doxorubicin treatment.

As active caspase‐1 has been reported to cleave GSDMD to generate GSDMD‐NT (32 kDa) in macrophages to induce pyroptosis,^15^ we next assessed whether cisplatin or doxorubicin induced caspase‐1‐mediated cleavage of GSDMD. Unexpectedly, although full‐length GSDMD (GSDMD‐FL) was decreased to produce a fragment (around 48‐50 kDa) in response to cisplatin or doxorubicin in RAW 264.7 cells, GSDMD‐NT was undetectable (Figure 2 A); in BMDMs, decrease in neither GSDMD‐FL nor GSDMD‐NT was detected (Figure 2 C), suggesting that GSDMD might not be involved in this process.

Recent studies revealed that beyond GSDMD, macrophages express another member of gasdermin family—GSDME, which can be cleaved by activated caspase‐3 to generate GSDME‐NT (37 kDa) that possesses pore‐forming activity to mediate secondary necrosis/pyroptosis.^5^, ^6^ Thus, we assessed whether GSDME was cleaved in macrophages treated with these chemotherapeutic drugs. Western blotting showed that concurrent with caspase‐3 activation, GSDME was cleaved and GSDME‐NT was dose‐dependently generated in both RAW 264.7 cells and BMDMs upon cisplatin or doxorubicin treatment (Figure 2 A‐D). The levels of GSDME‐NT were correlated with the percentages of necrosis revealed by PI staining (Figure 1 A‐F). Taken together, these results suggested that cisplatin‐ and doxorubicin‐induced secondary necrosis in mouse macrophages was probably mediated by the caspase‐3/GSDME axis.

### 3.3 Blockade of apoptotic caspases diminished GSDME cleavage and secondary necrosis in macrophages

**Figure 3:**
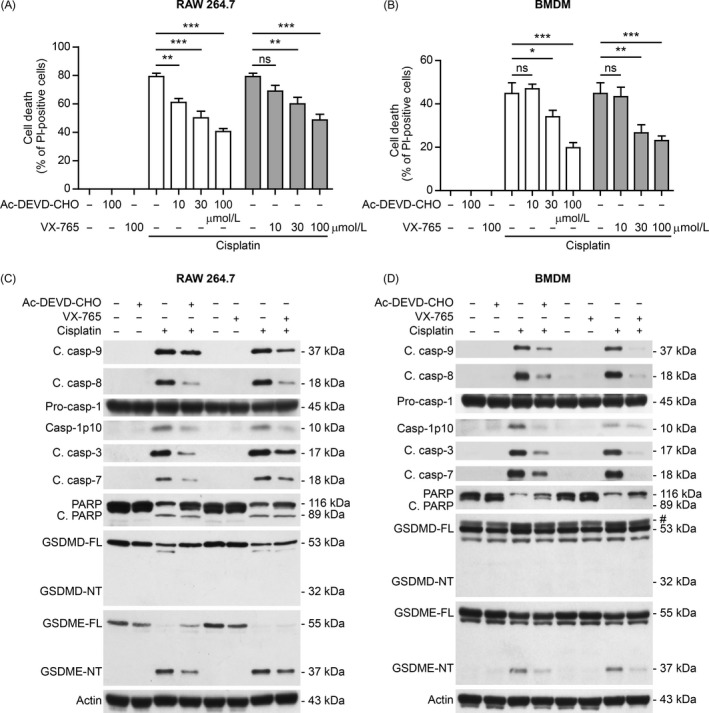
Inhibition of caspase‐3 and caspase‐1 attenuated cisplatin‐induced necrosis in mouse macrophages. A,B, RAW 264.7 cells (A) and bone marrow‐derived macrophages (BMDMs) (B) were pre‐treated with gradient doses of Ac‐DEVD‐CHO (caspase‐3 inhibitor) or VX‐765 (caspase‐1 inhibitor) for 1 h, followed by incubation with indicated dose of cisplatin (30 μmol/L) for 16 h. Necrosis was assayed by propidium iodide (PI) staining and observed with fluorescence microscopy. PI‐positive cells in 5 randomly chosen fields each containing ~100 cells were quantified. Data are shown as mean ± SD (n = 5). *P < .05; **P < .01; ***P < .001; ns, not significant. C,D, Western blot analysis of indicated proteins in RAW 264.7 cells (C) and BMDMs (D) were pre‐treated with Ac‐DEVD‐CHO (100 µmol/L) or VX‐765 (100 µmol/L) for 1 h and then incubated with cisplatin (30 µmol/L) for 16 h. Actin was used as a loading control. GSDME‐NT, GSDME N‐terminal fragment; GSDME‐FL, full‐length GSDME; C. casp, cleaved caspase

To confirm the role of caspase‐3 in chemotherapy agent‐induced necrosis, we next sought to explore whether pre‐treatment of caspases‐3 inhibitor Ac‐DEVD‐CHO (DEVD) could block the necrosis in mouse macrophages upon cisplatin treatment. DEVD dose‐dependently inhibited cisplatin‐induced necrosis both in RAW 264.7 cells and in BMDMs (Figure 3 A,B). It is yet unknown why DEVD could only suppress 50% of necrotic cell death; one possible reason is that other caspase‐3‐independent mechanism might have been involved in this process. Consistent with inhibition of necrosis, the activation of caspase‐3 and other caspases were also suppressed by DEVD, leading to decreased cleavage of PARP (suggestive of decreased apoptosis) and GSDME. Notably, GSDMD cleavage and production of the fragment (48‐50 kDa) were also reduced by DEVD in RAW 264.7 cells, suggesting that caspase‐3 had been involved in this process; however, no GSDMD‐NT was observed either in the presence or in the absence of DEVD. These results indicated that active caspase‐3 was responsible for the production of GSDME‐NT, which correlated with secondary necrosis in mouse macrophages.

As previous studies have shown that caspase‐1 can cleave and activate caspase‐3 and caspase‐7,^16^, ^17^, ^18^ we also sought to assess whether caspase‐1 inhibitor VX‐765 could suppress cisplatin‐induced necrosis and GSDME‐NT generation. VX‐765 dose‐dependently inhibited cisplatin‐induced necrosis in RAW 264.7 cells and BMDMs (Figure 3 A,B). Consistent with decreased necrosis, VX‐765 pre‐treatment also reduced the generation of GSDME‐NT and cleavage of PARP in RAW 264.7 cells and BMDMs (Figure 3 C,D). In line with this, we observed that cleaved caspase‐3 was markedly reduced by VX‐765 treatment in RAW 264.7 cells and BMDMs. Similar to DEVD treatment, VX‐765 also reduced GSDMD cleavage and production of the fragment (48‐50 kDa) in RAW 264.7 cells but not in BMDMs, but no GSDMD‐NT was detected either in the presence or in the absence of VX‐765 (Figure 3 C,D). These results indicated that caspase‐3‐mediated generation of GSDME‐NT, but not GSDMD‐NT, was associated with cisplatin‐induced secondary necrosis in mouse macrophages.

### 3.4 Knockdown of GSDME attenuated cisplatin‐ and doxorubicin‐induced secondary necrosis in macrophages

**Figure 4:**
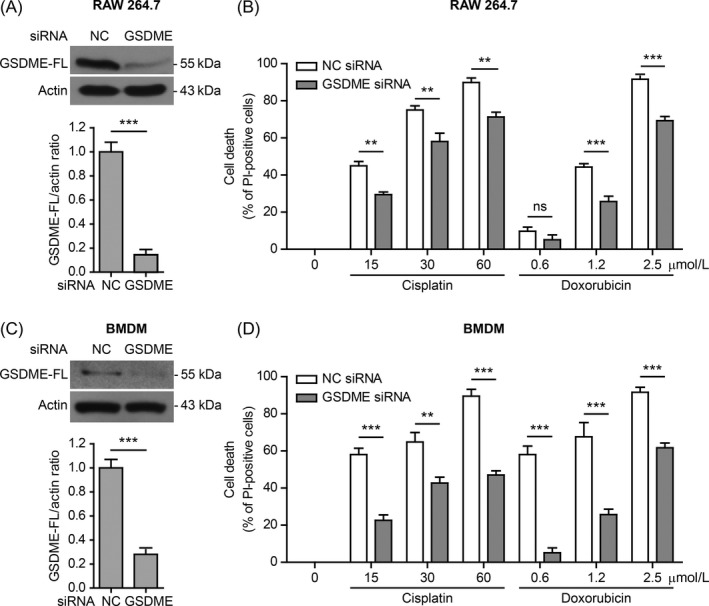
GSDME knockdown attenuated cisplatin or doxorubicin‐induced necrosis in macrophages. RAW 264.7 cells (A,B) and bone marrow‐derived macrophages (BMDMs) (C,D) were transfected with negative control (NC) siRNA or GSDME siRNA for 48 h, respectively. The cells were then treated with indicated doses of cisplatin and doxorubicin for 16 h. a, c Knockdown efficiency of GSDME in RAW 264.7 cells (A) and BMDM (C) was analysed by Western blotting. Actin was recruited as a loading control. Histograms show the amounts of GSDME levels relative to actin (n = 3). B,D, Necrosis in RAW 264.7 cells (B) and BMDMs (D) was measured by propidium iodide staining together with fluorescence microscopy. PI‐positive cells in 5 randomly chosen fields each containing ~100 cells were quantified. Data are shown as mean ± SD (n = 5). **P < .01; ***P < .001; ns, not significant

To further corroborate the role of GSDME in chemotherapy agent‐induced secondary necrosis, we knocked down its expression by siRNA. Western blotting showed that the knockdown yield of GSDME was ⁓80% in RAW 264.7 cells (Figure 4 A). Necrosis was significantly decreased after GSDME knockdown (Figure 4 B). Similar results were observed in BMDMs after GSDME knockdown (Figure 4 C,D). These observations indicated that GSDME was, at least partly, responsible for chemotherapeutic agent‐induced secondary necrosis in mouse macrophages.

### 3.5 Peritoneal macrophages were depleted by cisplatin administration accompanied by GSDME cleavage in vivo in mice

**Figure 5:**
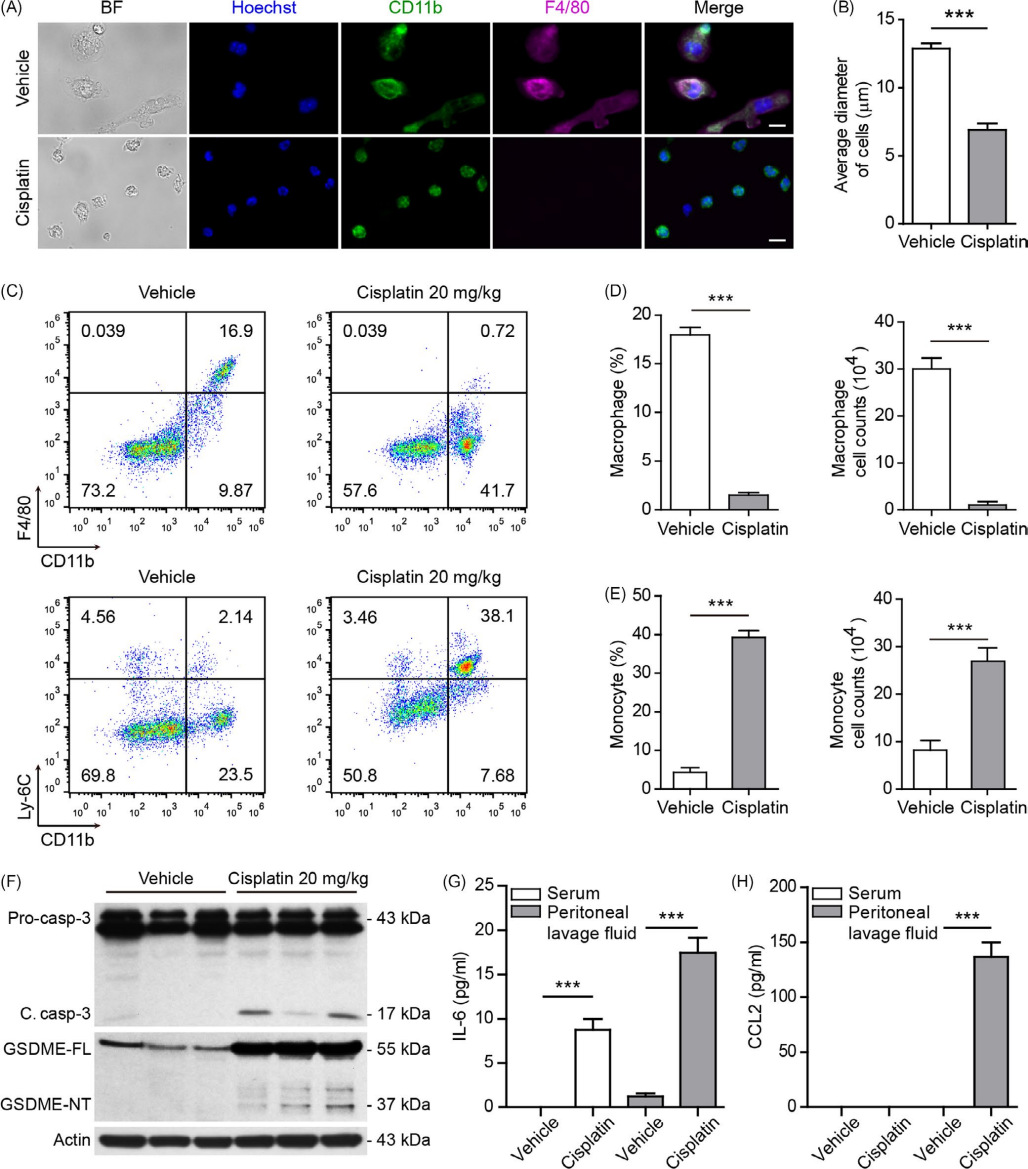
Cisplatin administration depleted peritoneal macrophages, activated caspase‐3 and caused cleavage of GSDME in vivo. A, Mice were administered (ip) with cisplatin or vehicle once. After 16 h, peritoneal exudate cells were isolated and seeded in glass‐bottom culture dishes for 4 h. The attached cells were fixed and stained with antibodies against CD11b and F4/80. Nuclei were revealed by Hoechst 33342. Representative images captured by fluorescence microscopy are shown. Scale bars, 10 μm. B, Quantification of average cell diameters in each group. Data are shown as mean ± SD (n = 50). C, Flow cytometric analysis of macrophages (CD11b^+^F4/80^+^) and monocytes (CD11b^+^Ly‐6C^+^) in the peritoneal cavity after bacterial infection. One representative set of flow cytometric dot plots are shown. CD11b^+^F4/80^+^ cells (D) and CD11b^+^Ly‐6C^+^ cells (E) in (C) were quantified by their percentages times the total peritoneal cell numbers (determined by a hemocytometer), respectively. Data are expressed as mean ± SD (n = 3). ***P < .001. F, Western blot analysis of caspase‐3 and GSDME in the peritoneal exudate cells in mice (n = 3). The blots of three individual mice in each group were displayed. Actin was used as a loading control for cell lysates. G,H, IL‐6 (G) and CCL2 (H) in mouse serum and peritoneal lavage fluids were measured by cytometric bead array. Data are shown as mean ± SD (n = 3). ***P < .001

To confirm the in vitro data of chemotherapy drugs in inducing GSDME‐mediated necrosis in macrophages, we sought to explore whether cisplatin was toxic to mouse peritoneal macrophages in vivo. Mice were intraperitoneally injected with cisplatin or vehicle once, and peritoneal exudate cells and serum were collected 16 hours after treatment. As shown in Figure 5 A, the expression of F4/80 (a marker of mature macrophages), which was detected in the peritoneal exudate cells from vehicle‐treated mice, was barely detectable in the counterparts of cisplatin‐treated mice, indicating that the depletion of mature peritoneal macrophages by cisplatin. Notably, the attached cells from cisplatin‐treated mice expressed CD11b (a marker of myeloid cells) but not F4/80, and their sizes (~7 μm) were drastically decreased as compared to the sizes (~12 μm) of vehicle group (Figure 5 B), suggesting that myeloid cells had been newly recruited. Flow cytometric analysis verified that the percentages, and absolute counts of mature peritoneal macrophages (CD11b^+^F4/80^+^) were markedly decreased in cisplatin‐treated group compared with vehicle‐treated group (Figure 5 C,D). In contrast, the proportions and absolute counts of inflammatory macrophages (CD11b^+^Ly‐6C^+^) were markedly increased in cisplatin‐treated group (Figure 5 C,E). These results suggested that the mature peritoneal macrophages had been depleted by cisplatin administration and that a large number of inflammatory macrophages had been newly recruited into the peritoneal cavity of mice.

Based on the aforementioned in vitro results, we hypothesized that the peritoneal macrophages might have undergone GSDME‐mediated secondary necrosis in vivo after cisplatin treatment, leading to an inflammatory milieu to recruit monocytes. To this end, Western blotting was utilized to assess the cleavage of GSDME and activation of caspase‐3. Indeed, cleaved caspase‐3 was detected in the peritoneal exudate cells from cisplatin‐treated mice but not in cells from vehicle‐treated group (Figure 5 F). Concurrent with caspase‐3 activation, GSDME was cleaved to generate GSDME‐NT in cisplatin‐treated cells. This suggested that cisplatin treatment might be associated with GSDME‐mediated secondary necrosis (an inflammatory form of cell death) in the peritoneal cavity to induce inflammation, which prompted us to assay inflammatory cytokines in the peritoneal lavage fluids and serum of mice. Cytometric bead array analysis showed that interleukin‐6 (IL‐6) levels in the peritoneal lavage fluids and serum were detected after cisplatin administration, whereas it was barely detectable in vehicle‐treated mice (Figure 5 G). CCL2, an chemoattractant of monocytes,^19^, ^20^ was markedly increased in the peritoneal lavage fluids, but not in the serum, of the mice administered with cisplatin (Figure 5 H), which was consistent with the recruitment of monocytes in their peritoneal cavity (Figure 5 E). Together, these results suggested that cisplatin induced depletion of peritoneal macrophages, which was associated with caspase‐3/GSDME‐mediated secondary necrosis in mice.

### 3.6 Cisplatin decreased the survival of mice infected with *E coli*

**Figure 6:**
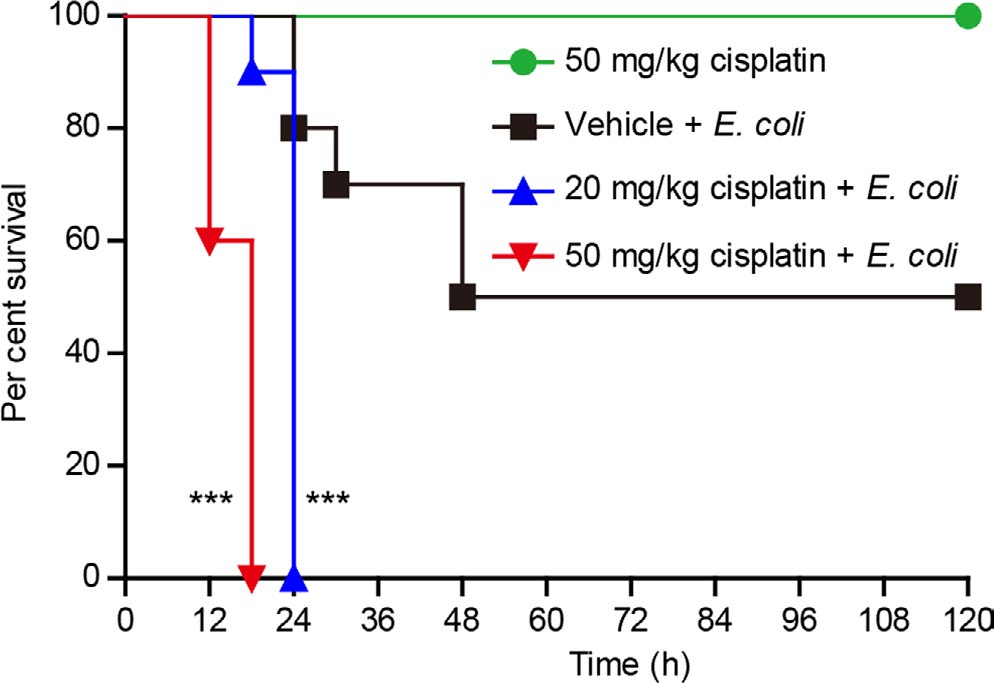
Cisplatin administration reduced the survival of mice with bacterial infection. Mice were administered (ip) with cisplatin or vehicle once 3 h before injection (ip) with viable *E coli* (1.5 × 10^9^ CFU/mouse). Mouse survival was monitored every 6 h for five consecutive days. Kaplan‐Meier survival curves were used to analyse the data (10 mice per group). The significance was evaluated by the log‐rank (Mantel–Cox) test. ***P < .001

As peritoneal macrophages act as tissue‐resident macrophages playing important roles in defending against infection and in maintaining peritoneal homeostasis,^10^ cisplatin‐induced depletion of them might impair the innate immunity in the peritoneal cavity. To test this hypothesis, we adopted a mouse model of bacterial infection with or without cisplatin administration. About 50% of the vehicle‐treated mice were able to survive the period of observation, whereas no cisplatin‐treated mice survived 24 hours (Figure 6). These results suggested that cisplatin‐induced depletion of peritoneal macrophages had contributed to the acute animal death.

## 4 DISCUSSION

The toxicity of chemotherapeutic drugs to normal organs is one major limitation for their clinic application against cancers. Recent studies have identified GSDME expression as an important factor in mediating the toxicity of chemotherapeutic agents to normal tissues by inducing pyroptosis.^5^ Macrophages also express GSDME protein,^6^ but whether they undergo secondary necrosis/pyroptosis upon exposure to chemotherapeutic drugs is incompletely understood. We in this study revealed that cisplatin and doxorubicin exerted their toxicity to mouse macrophages by inducing secondary necrosis, which was at least partly mediated by the caspase‐3/GSDME axis. Thus, chemotherapy drug‐induced secondary necrosis in macrophages is likely a contributing factor to the adverse toxicity of these chemotherapeutic drugs.

Both GSDMD and GSDME are the members of gasdermin family.^15^ Notably, GSDME is often down‐regulated or even silenced in cancer cells but is expressed in many normal tissues.^7^, ^8^, ^9^ In humans, *GSDME* gene is highly expressed in the lung, kidney, testis and placenta, while it is moderately expressed in the heart, pancreas, stomach, small intestine and brain.^7^, ^9^ Consistent with high or moderate GSDME expression in respective organs, nephrotoxicity and neurotoxicity constitute the major side effects of cisplatin, while cardiotoxicity and neurotoxicity are the major adverse effects of doxorubicin in humans.^1^ In line with high GSDME expression in macrophages, our present study revealed that both cisplatin and doxorubicin exhibited robust toxicity by inducing secondary necrosis in macrophages. This secondary necrosis at least partly relied on caspase‐3‐mediated cleavage of GSDME‐NT as blockade of caspase‐3 or knockdown of GSDME significantly reduced such necrosis, consistent with the finding that caspase‐3 is the only caspase that cleaves GSDME.^5^ Collectively, chemotherapy drugs like cisplatin and doxorubicin can exert their toxicity to mouse macrophages by inducing GSDME‐mediated necrosis.

As DNA‐damaging agents, cisplatin and doxorubicin have been shown to activate both intrinsic (caspase‐9) and extrinsic (caspase‐8) apoptotic pathways.^4^ In line with previous studies, we found that these two drugs did activate caspase‐8/caspase‐9 in mouse macrophages leading to the activation of downstream caspase‐3/caspase‐7. Active caspase‐3 in turn cleaved GSDME to generate GSDME‐NT. On the other hand, the inflammatory caspase‐1 was also activated by cisplatin and doxorubicin. Although it is unknown how caspase‐1 had been activated by the drugs, the active caspase‐1 had contributed to the activation of caspase‐3 as inhibition of caspase‐1 activity by VX‐765 suppressed the generation of cleaved caspase‐8/caspase‐9, as well as downstream caspase‐3/caspase‐7, consistent with previous studies showing that caspase‐1 can cleave caspase‐3/caspase‐7.^16^, ^17^, ^18^ Surprisingly, no GSDMD‐NT was detected in our study given that caspase‐1 was activated, which has been reported to cleave GSDMD.^18^ One possible explanation for this is that the high levels of activated caspase‐3 might have overridden this process. An alternative possibility is that activated caspase‐3/caspase‐7 can inactivate GSDMD by cleaving it at a distinct site (D88 in mice and D87 in humans) leading to the formation of other GSDMD fragments instead of the N‐terminal region,^17^ which might not be detected by the antibody (detecting the 53 and 32 kDa bands) used in this study. Although VX‐765 used in this study is a superior caspase‐1 inhibitor than Ac‐YVAD‐cmk,^21^ further research using caspase‐1 knockout cells is warranted to verify the role of caspase‐1 in mediating caspase‐3 activation in macrophages. Together, chemotherapy drug‐induced caspase‐3 activation, either by apoptotic caspase‐8/caspase‐9 or by probably inflammatory caspase‐1, can trigger secondary necrosis concurrent with apoptosis in cells expressing GSDME protein.

As chemotherapy drug‐induced secondary necrosis is a lytic form of cell death,^5^, ^6^ macrophages may be reduced or depleted by such drugs. In this study, we found that mouse peritoneal macrophages were depleted after in vivo cisplatin treatment and that the activation of caspase‐3 and GSDME among these cells during the cisplatin treatment indicated induction of secondary necrosis, yet we cannot exclude the direct toxicity of cisplatin on the macrophages. Given the important roles of the peritoneal macrophages acting as tissue‐resident innate immune cells,^10^ such depletion may impair the innate immunity against bacterial infections. Indeed, cisplatin‐treated mice all succumbed to a sub‐lethal dose of intraperitoneal *E coli* infection, suggesting that chemotherapy drugs have impaired the first‐line defence of the innate immunity. Although newly recruited monocytes had been markedly increased, they may still need time to differentiate into mature macrophages and whether the differentiated cells (if there were) can functionally act as the peritoneal resident macrophages remains unclear.^10^ Given that GSDME is expressed in various normal tissues (eg spleen, kidney, intestine and testicle),^5^ the exact role of GSDME in mediating cisplatin toxicity awaits future investigation in mice with macrophage‐specific knockout of GSDME. On the other hand, lytic cells release various inflammatory cellular components, leading to inflammation responses.^22^ In line with this, cisplatin administration had increased the levels of inflammatory cytokines such as IL‐6 in the peritoneal cavity and blood of mice, suggestive of the induction of low‐level systemic inflammation. Such chemotherapy drug‐induced inflammation has been extensively investigated and may lead to the failure of chemotherapy and metastasis.^23^ Taken together, chemotherapy drugs may not only exert detrimental influences on the innate immune cells like macrophages but also induce systemic inflammation that could hinder their therapeutic effects or even promote metastasis.

However, recent studies showed that GSDME is not involved in regulating secondary necrosis in human T cells and monocytes^24^ and that GSDME is dispensable for the secondary necrosis that follows NLRC4‐mediated apoptosis in macrophages,^25^ suggesting that other as‐yet‐unidentified caspase‐3 substrates may exist in caspase‐3‐mediated necrosis. Whether such as‐yet‐unidentified substrates participate in chemotherapy drug‐induced necrosis in macrophages warrants future investigation.

In summary, we showed that chemotherapy drugs, such as cisplatin and doxorubicin, induced secondary necrosis concurrent with apoptosis in mouse macrophages. This form of necrosis was likely mediated by the caspase‐3/GSDME axis. Induction of secondary necrosis of macrophages may not only have a role in depleting tissue‐resident macrophages thus impairing the innate immunity, but also might foster a systemic inflammation milieu that weakened the chemotherapeutic effects on cancers. The chemotherapy drug‐induced secondary necrosis in macrophages therefore constitutes another form of toxicity, and targeting GSDME‐mediated necrosis may be a novel avenue to mitigate the side effects of chemotherapy drugs.

## CONFLICT OF INTEREST

The authors have no conflicts of interest to declare.
